# Cross-Talk between Apolipoprotein E and Cytokines

**DOI:** 10.1155/2011/949072

**Published:** 2011-06-28

**Authors:** Hongliang Zhang, Li-Min Wu, Jiang Wu

**Affiliations:** ^1^Department of Neurology, The First Hospital of Jilin University, Jilin University, 130021 Changchun, China; ^2^Division of Neurodegeneration and Neuroinflammation, Department of Neurobiology, Care Sciences and Society, Karolinska Institute, Karolinska University Hospital Huddinge, Novum, plan 5, 141 86 Stockholm, Sweden

## Abstract

Apolipoprotein E (apoE) is a multifunctional glycosylated protein characterized by its wide tissue distribution. Despite its importance in lipid transport and atherosclerosis pathogenesis, apoE is associated with neurodegenerative disorders such as Alzheimer's disease (AD) and Parkinson disease, and autoimmune disorders such as multiple sclerosis and psoriasis. Among others, the role of apoE in modulating inflammation and oxidation is crucial in elucidating the risk factors of the above diseases since the function of apoE is closely linked with both proinflammatory and antiinflammatory cytokines. Moreover, apoE modulates inflammatory and immune responses in an isoform-dependent manner. Correspondingly, inflammatory cytokines can either upregulate or downregulate the production of apoE in various tissue types. However, studies on the interactions between apoE and cytokines occasionally yield conflicting results, highlighting the complex roles of apoE and cytokines in various disorders. The present paper summarizes the current knowledge about the cross-talk between apoE and cytokines, with emphasis on the effects of apoE on the Th1/Th2 balance.

## 1. Introduction

**Figure 1:**
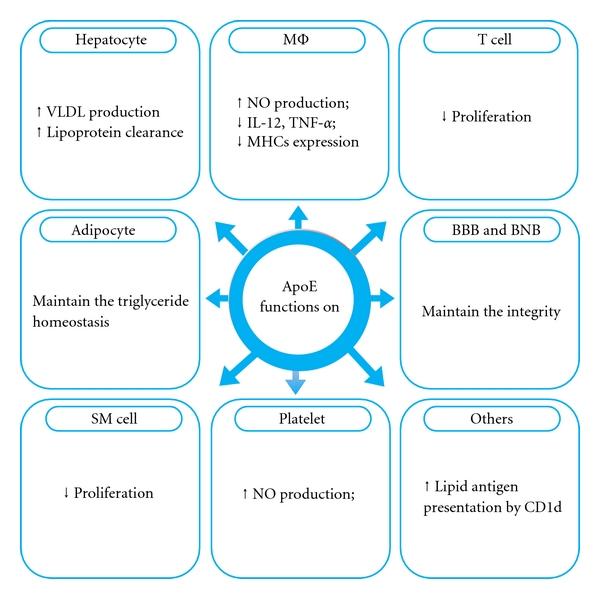
Schematic illustration of the biological properties of apoE. ApoE acts on adipocytes to maintain the triglyceride homeostasis; acts on hepatocytes to promote very low density lipoprotein (VLDL) production and lipoprotein clearance; modulates the functions of macrophages (MΦ); suppresses the proliferation of T cells; maintains the integrity of blood-brain barrier (BBB) and blood-nerve barrier (BNB); inhibits the proliferation of smooth muscle (SM) cells; upregulates the production of nitric oxide (NO) of platelets; facilitates the presentation of lipid antigen by CD1 molecules to natural killer T (NKT) cells; and so forth. ↑ denotes upregulation or induction; ↓ downregulation or inhibition; IL denotes interleukin; TNF-*α* denotes tumor necrosis factor alpha; MHC denotes major histocompatibility complex.

Apolipoprotein E (apoE) was first discovered in 1970s and identified as a component of triglyceride-rich lipoprotein complexes. Since then, apoE has been widely studied in lipid metabolism, cardiovascular diseases [1], and neurodegenerative disorders such as Alzheimer's disease (AD) [2] and Parkinson disease (PD). In addition to its well-established role in lipid transportation and atherosclerostic pathogenesis, apoE bears immunomodulatory properties and is associated with multiple sclerosis (MS) [3] and psoriasis [4]. ApoE can modulate the functions of macrophages, suppress the proliferation of T cells, maintain the integrity of blood-brain barrier (BBB) and blood-nerve barrier (BNB), inhibit the proliferation of smooth muscle (SM) cells, upregulate the production of nitric oxide (NO) of platelets, facilitate the presentation of lipid antigen by CD1 molecules to natural killer T (NKT) cells, and so forth, (Figure 1). Cytokines are crucial in human inflammatory and autoimmune disorders and apoE might affect these disorders through interacting with cytokines.

## 2. A General View of Human ApoE, Its Polymorphism, and Its Receptors

**Figure 2:**
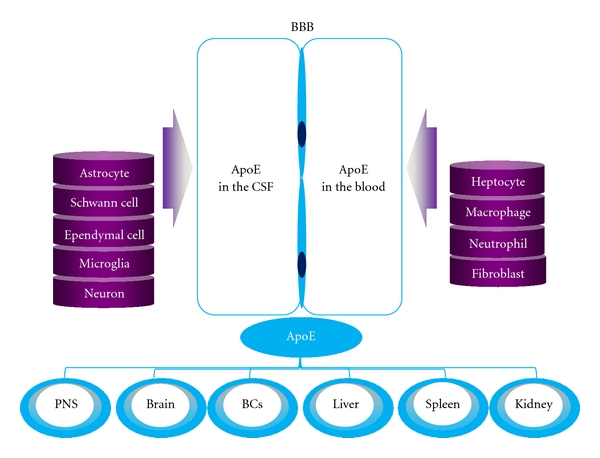
The synthesis and secretion of apoE. Hepatic parenchymal cells are the principal apoE producing cells, presumably accounting for 60% to 75% of apoE in plasma, followed by astrocytes, which are the main apoE producing cells in the nervous system. ApoE mRNA can also be found in spleen, lung, kidney, peripheral nerves, muscular tissue, adrenal, ovary and testis, and so forth. In the nervous system, apoE mRNA is present in astrocytes, nonmyelinating Schwann cells, ependymal cells, microglia, and neurons, and so forth. BCs denotes blood cells; PNS denotes peripheral nervous system; CSF denotes cerebrospinal fluid.

Human apoE is a 34.2 kDa glycoprotein with 299 amino acid residues. ApoE is produced by liver, brain, spleen, kidney, lung and muscle tissues, and so forth. Hepatic parenchyma cells produce 2/3 to 3/4 of the apoE in plasma [5]. In the nervous system, apoE mRNA is present in astrocytes, microglia, Schwann cells (SCs), neurons, and so forth, (Figure 2) [6]. There are three major isoforms of human apoE (apoE2, apoE3, apoE4) encoded by the *ε*2, *ε*3, and *ε*4 alleles on chromosome 19q13. Differences in the amino acid residues (positions 112/158) of apoE distinguish the apoE2 (Cys/Cys), apoE3 (Cys/Arg), and apoE4 (Arg/Arg) isoforms. The APOE*ε* alleles show a peculiar distribution throughout the world [7]; the APOE*ε*3 allele is the most frequent in populations with a long-established agricultural economy, whereas the APOE*ε*4 allele is the ancestral allele, with a higher frequency in Pygmies, Khoi San, Papuans, Lapps, and some Native Americans [8]. The frequency of the APOE*ε*4 allele also increases with latitude [9].

The structural polymorphism in apoE influences its conformation and bindings to lipoprotein particles [10] and cellular receptors [11]. ApoE4 preferentially binds to lower density lipoproteins and is associated with increased risk of atherosclerosis and neurodegenerative disorders, including AD. This binding preference is due to domain interaction (the presence of Arg-112 causes Arg-61 in the amino-terminal domain to interact with Glu-255 in the carboxyl-terminal domain) (Table 1). Additionally, physiological properties of apoE, such as antioxidant [12], antiapoptotic [13], immunomodulatory [14], and atheroprotective capacities [15] are significantly influenced by apoE polymorphism.

ApoE exerts its biological functions, especially lipid transportation, by binding to its receptors, the low-density lipoprotein receptor (LDLR) family. The LDLR family includes LDLR, LDLR-related protein 1 (LRP1), very low density lipoprotein receptor (VLDLR), apoE receptor (apoER)2, megalin (also known as LRP2) and LRP1B, and so forth, [16]. As the prototype of this family, LDLR is the main receptor for cholesterol metabolism [17], to which apoE3 and apoE4 bind with 50-fold greater affinity than apoE2 [18]. ApoE is essential for the metabolism of triglyceride-rich lipoprotein constituents. The interaction of apoE and LDLR determines the removal of apoE-rich lipoproteins (VLDL, chylomicron remnants, intermediate density lipoproteins), and the homeostasis of cholesterol and triglycerides. Besides direct signal transduction, the LDLR family functions as signal modulators and integrators in several signaling pathways [19]. 

There is only one isoform of apoE in rodents. Murine apoE preferably associates with HDL, and its clearance is mainly through LDLR [20].

## 3. ApoE, Lipids, and Infections

The distribution of lipids among different body compartments occurs by means of different lipoprotein particles in the blood circulation [21]. Host responses to infections involve changes in lipid levels and lipid metabolism in the plasma. These plasma lipid changes might be mediated by three cytokines interleukin (IL)-1, IL-6, and tumor necrosis factor (TNF)-*α*, which induce elevated levels of triglycerides and VLDL [22], decreased levels of cholesterol, HDL and LDL [23, 24] or increased levels of cholesterol in the serum [25], depending on the nature of the infectious agents.

Lipoproteins and lipids present in the serum may contribute to the host innate immunity against pathogens [26]. Studies using apoE deficient mice confirmed the role of apoE in host susceptibility to endotoxemia and *Klebsiella pneumoniae* infection [27], while transgenic (Tg) mice expressing human APOE*ε*3 and APOE*ε*4 genes revealed an isoform-specific effect of apoE on the proinflammatory response to lipopolysaccharide (LPS) [28]. Infection of apoE knockout (KO) mice with *Listeria monocytogenes* or *Klebsiella pneumoniae *leads to an increased susceptibility to death as well as increased serum levels of TNF-*α* as compared with wild-type (WT) mice [29, 30]. Binding of bacterial endotoxin to either HDL, LDL, or VLDL results in a redirection of endotoxin uptake from Kupffer cells to parenchymal hepatocytes where the endotoxin is deactivated [30]. ApoE facilitated sepsis-induced mortality in a dose-dependent manner and increased natural killer T-(NKT)-cell proliferation in the spleen [31]. ApoE deficient mice were markedly more susceptible to tuberculosis, evidenced by 100% mortality within 4 weeks of infection with tuberculosis [32].

ApoE *ε*4/*ε*4 genotype was associated with an aggravated disease course of acquired immunodeficiency syndrome (AIDS), especially with accelerated progression to death. Compared with apoE3, apoE4 enhanced human immunodeficiency virus (HIV) fusion/cell entry of both R5 and X4 HIV strains *in vitro* [33]. ApoE4 competes less efficiently than the other isoforms for entry into neuronal cells through heparan sulphate proteoglycan proteoglycans, which are involved in HIV attachment and entry into cells [34]. HIV produces a chronic viral infection in the central nervous system (CNS) that elicits chronic glial activation and cytokines overproduction. Corder et al. showed that HIV-infected subjects with an APOE*ε*4 allele had excess peripheral neuropathy [35]. As a well-known risk factor for AD [2], an *ε*4 allele along with the presence of herpes simplex virus (HSV) 1 in the brain confers an increased risk of developing to AD [36, 37]. 

The production of infectious hepatitis C virus (HCV) was significantly reduced by the knockdown of apoE. The genotypes carrying the APOE*ε*2 were associated with the equivalent of a 3–5-fold reduction in the risk of chronic HCV infection [38]. Another study, however, showed that the expression of the apoE2 resulted in poorer recovery of infectious HCV than did apoE3 and apoE4 isoforms [39]. Either subjects without APOE*ε*3 or with a single APOE*ε*3 with high serum cholesterol are prone to a better prognosis [40]. ApoE gene polymorphism was also associated with hepatitis B virus (HBV) infection, and the *ε*2 allele, compared with the other alleles, showed positive correlation with different HBV infection models [41].

## 4. Effects of ApoE on the Production of Cytokines and the Maintenance of the T Helper (Th)1/Th2 Balance

ApoE suppressed inflammation through VLDLR or apoER2 in macrophages by converting proinflammatory M1 to the antiinflammatory M2 phenotype [42]. Exogenous apoE suppressed LPS and polyinosine-polycytidylic acid (PIC, a double-stranded RNA that serves as a viral mimetic and a Toll-like receptor (TLR)3 agonist) induced secretion of IL-6, IL-1*β* and TNF-*α* by RAW 264.7 cells via repressing TLR-agonist-induced phosphorylation of c-Jun N-terminal kinase (JNK) and c-Jun. ApoE peptide (141–155)2 inhibits LPS- and PIC-induced macrophage inflammatory responses in a similar manner [43]. ApoE also reduces inflammatory signaling in astrocytes and microglia in response to proinflammatory stimuli [44, 45].

**Figure 3:**
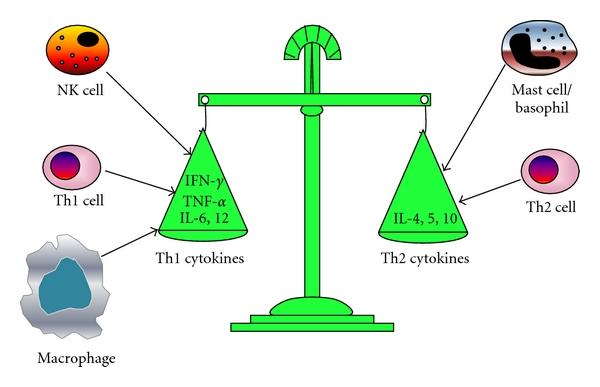
The T helper (Th)1/Th2 balance. Th1 cytokines, characterized by the production of interferon (IFN)-*γ* and interleukin (IL)-6, are involved in cellular immunity and immunoglobulin class switching to the IgG2a isotype, whereas Th2 cytokines, characterized by IL-4 and IL-10, are mainly associated with humoral immunity and immunoglobulin class switching to IgG1 and IgE. Th1 cells are the primary Th1 cytokine producing cells. Besides, natural killer (NK) cells and macrophage also contribute to the production of Th1 cytokines. Similarly, Th2 cells produce the most Th2 cytokines in addition to mast cells and basophils. TNF-*α* denotes tumor necrosis factor alpha.

Th precursor (THP) cells can differentiate into Th1, Th2, or Th0 cells. Th0 cells can differentiate to Th1 and Th2 subpopulations depending primarily on the cytokines provided exogenously or from dendritic cells. Th1 cells are involved in cellular immunity and immunoglobulin class switching to the IgG2a isotype, whereas Th2 cells are mainly associated with humoral immunity and immunoglobulin class switching to IgG1 and IgE. Moreover, Th1 cells promote the induction of complement-fixing, opsonizing antibodies and of antibodies involved in antibody-dependent cell cytotoxicity, for example, IgG2a in mice. Th1 cytokines include interferon (IFN)-*γ*, IL-12, and TNF-*α*. They can activate macrophages to produce reactive oxygen intermediates and NO, stimulate their phagocytic functions, and enhance their antigen presenting capacity by upregulating major histocompatibility complex (MHC) class molecules. Th2 cytokines include IL-4, IL-5, IL-10, and IL-13, which provide potent help for B-cell activation, immunoglobulin class switching to IgE and IgG1 in the mouse, and downregulation of macrophage activation (Figure 3). 

Imbalance of the Th1/Th2 can result in autoimmune disorders. In experimental autoimmune neuritis (EAN), an animal model of human Guillain-Barré syndrome (GBS), Th1 cytokines predominate and mediate inflammatory damage to the peripheral nerves, whereas Th2 cytokines are associated with recovery from the disease [46–48]. Proinflammatory cytokines such as IL-1*β*, IL-6, IL-12, IL-17, IL-18, IL-23, TNF-*α*, IFN-*γ*, and macrophage inhibitory factor (MIF) significantly contribute to disease development of EAN by recruiting effector cells to the peripheral nervous system (PNS) and by enabling *in situ* release of other products toxic for SCs and myelin such as free radicals, oxygen intermediates, and NO [46, 47, 49–51]. Antiinflammatory cytokines from Th2 cells such as IL-4 and IL-10 may suppress the disease by playing an important antiinflammatory role [52, 53]. The balance of functionally distinct T-cell subsets between Th1 and Th2 has a direct relevance to autoimmune disorders.

Recently, the Th1/Th2 paradigm has been challenged, following the discovery of a third subset of effector Th cells that produce IL-17 (termed Th17 cells) and exhibit effector functions distinct from Th1 and Th2 cells. Th17 cells are potent inducers of tissue inflammation and have been associated with the pathogenesis of many autoimmune diseases and rheumatic disorders [54]. IL17-producing Th17 cells have been found to play an important role in many autoimmune diseases including experimental autoimmune encephalomyelitis (EAE), an animal of MS, while thymic regulatory T (Treg) cells have a suppressive role, although their roles in EAN and GBS need further investigation [55]. Increased mRNA level of IL-1*β*, IL-2, IL-6, IFN-*γ*, intercellular adhesion molecule (ICAM)-1, vascular cell adhesion molecule (VCAM)-1, monocyte chemoattractant protein (MCP)-1, nuclear factor (NF)*κ*B (p65) and inhibitory (I)*κ*B-*α* and decreased mRNA level of IL-4, IL-10, and granulocyte-macrophage colony-stimulating factor (GM-CSF) in apoE KO mice compared with WT mice imply that lacking apoE promotes Th1 immune responses by changing these cytokines [56]. IL-6, IL-12, TNF-*α*, and IFN-*γ* were upregulated to a significantly greater level in apoE-deficient mice than in WT mice at both the mRNA and protein levels in response to LPS administration. ApoE selectively regulates TLR4- and TLR3-mediated signaling of IL-12 production and apoE may suppress the Th1 immune response by modulating IL-12 production [57]. Macrophages from apoE-deficient mice stimulated by exogenous antigens are more effective in upregulating MHC class *Ⅱ* molecules and CD80, with elevated secretion of IFN-*γ* in responding T cells [58]. Severe hypercholesterolemia can induce a switch of autoimmune responses from Th1 to Th2 effector type in atherosclerotic apoE KO mice [59]. ApoE-deficient mice injected with LPS produced elevated levels of IL-1*β*, IL-6, and TNF-*α*, when compared with WT mice, and exogenous apoE could reverse this effect [60].

ApoE targets myeloid differentiation primary response gene (MyD)88 and interleukin-1 receptor-associated kinase (IRAK)1 activation whereby interrupting IL-1*β* and IL-18 signaling in vascular smooth muscle cells (VSMCs). As a consequence, apoE can inhibit VSMC activation in response to IL-18. IL-1*β* signaling intermediates NF*κ*B transactivation was inhibited by apoE in MyD88- and IRAK1- but not in TRAF6-transfected cells. ApoE can prevent IRAK1 phosphorylation and IRAK1-TRAF6 but not MyD88-IRAK1 complex formation [61]. 

NO is a principal effector of macrophage-mediated inflammatory/immune responses. In the mononuclear-phagocyte system, NO is formed enzymatically from L-arginine by inducible nitric oxide synthase (iNOS) [62]. Treatment of microglia and peripheral macrophages with apoE increased NO production stimulated by IFN-*γ* and LPS [63]. ApoE *per se*, however, seems unable alone to induce the production of either iNOS; it functions via alteration of arginine availability [64]. Furthermore, upon proinflammatory stimulation, peripheral macrophages from male APOE*ε*4 Tg mice could produce significantly higher levels of NO than from APOE*ε*3 Tg mice [65]. Similar results were also found in human studies [66]. The elevated NO production is coupled with an increased arginine uptake in male APOE*ε*4 Tg mice and is dependent on p38 mitogen-activited protein kinase (MAPK) [67], whereas it is not the case in female mice. Macrophages from female APOE Tg mice produce higher levels of NO than male ones, without any isoform-dependent differences [68]. 

SCs can secrete cytokines like IL-6 and IL-10 and actively participate in immune responses as facultative antigen presenting cells in the PNS [69]. The antigen presenting properties of SCs were enhanced in apoE KO mice, concomitant with downregulated production of intracellular IL-6 [70]. In the recovery stage of EAN, NO produced by SCs is pivotal for the termination of local immune responses by inducing apoptosis of effector T cells in the PNS [65]. ApoE may regulate local inflammation in the PNS via modulating cytokine and NO production. ApoE might act as an inhibitor for EAN by suppressing the Th1 response and shifting the Th1/Th2 to the Th2 direction. An increased susceptibility to EAN after upregulation of the autoreactive T-cell response to peripheral nerve component with higher levels of IFN-*γ*, IL-12, and TNF-*α* and lower levels of IL-10 was seen in apoE KO mice than WT mice [71]. 

Significantly increased secretion of Th17-related cytokines (IL-17 and IL-6) and expression of transcription factor retinoid-related orphan receptor gamma (ROR*γ*)t levels and obviously decreased number in Treg cells, secretion of Treg-related cytokines (TGF-*β*1) and expression of transcription factor (Foxp3) levels were found in apoE KO mice. Th17/Treg functional imbalance exists in apoE KO mice, suggesting a role of Th17/Treg imbalance in apoE functioning [72]. IL-17A was also found to be elevated in the plasma and tissues of apoE null mice [73].

COG133, a mimetic of apoE protein, when binding to a protein transduction domain creates COG112, an antennapedia-linked apoE-mimetic peptide, improves therapeutic effects on EAE through decreasing demyelination and diminishing the number of peripheral cells infiltrating into the spinal cord, as well as other inflammatory processes [74, 75]. Increased mRNA level of iNOS, TNF-*α*, IFN-*γ*, and IL-17 and decreased nuclear translocation of NF-*κ*B and I*κ*B kinase (IKK) activity in colitis models suggest that COG112 inhibits inflammation through the NF*κ*B pathway [76]. Furthermore, intravenous administration of a small apoE-mimetic peptide derived from the receptor-binding region of the apoE holoprotein (apoE-(133–149)) similarly suppressed both systemic and local brain inflammatory responses in mice after LPS administration [28]. However, different genetic basis determines modified immunological processes [77].

ApoE plays its nonlipid related roles by binding to cell surface receptors [78]. LRP is implicated to mediate the immunomodulatory effects of apoE, although there seems to be no difference in the binding affinity of apoE isoforms to LRP [79, 80].

## 5. Isoform-Dependent Effects of ApoE on the Production of Cytokines

ApoE suppressed the secretion of TNF-*α* and IL-1*β* in an isoform-specific fashion (E2 > E3 > E4) [67]. A markedly higher level of TNF-*α* was observed in supernatant of cultured macrophage derived from adult male APOE*ε*4 Tg mice compared with macrophages from APOE*ε*3 ones [81]. In the presence of LPS, apoE3-expressing cells produce less TNF-*α* and IL-6 than apoE2 and apoE4-expressing cells which might be related with the extracellular signal-regulated kinases (ERK)1/2 signaling pathways [82]. ApoE genotype pronouncedly affects the cellular immune response in stably transfected murine macrophages. In apoE4 versus apoE3 macrophage cell line cells higher levels of proinflammatory cytokines including TNF-*α* appeared evident followed by LPS stimulation [83]. Preincubation with human recombinant apoE blocked glial secretion of TNF-*α* in mixed neuron-glial cultures in response to LPS stimulation in a dose-dependent manner (apoE3 > apoE4). This effect was the greatest when apoE was preincubated for 24 hours and was not dependent on any direct effect of apoE on cell viability [84].

Twice as many apoE4 carriers were demented (30% versus 15%) or had peripheral neuropathy as compared with non-apoE4 carriers in the HIV infection [35]. If there are apoE isoform-dependent differences in the antiinflammatory function of apoE *in vivo*, such differences might in part explain the differential risk for AD caused by apoE isoforms. However, several studies demonstrated that exogenously applied apoE4 had robust proinflammatory activity in astrocytes and microglial cells [85, 86]. In mice receiving the basal diet without quercetin supplementation, levels of TNF-*α* in whole blood stimulated *ex vivo *with LPS were higher in apoE3 than apoE4 Tg mice [87].

ApoE displayed an isoform-specific effect on inflammation in primary adult microglia culture, with apoE4 the most potent to stimulate production of prostaglandin-E2 and IL-1*β* [88]. Activation of primary astrocytes from APOE targeted replacement (TR) mice with LPS led to genotype-dependent differences in cytokine secretion that were the greatest in APOE*ε*2 TR mice (APOE2 > APOE3 > APOE4) [89]. Activation of primary astrocytes from APOE TR mice with LPS led to APOE-dependent differences in cytokine secretion that were greatest in APOE*ε*2 TR mice and that were associated with differences in NF*κ*B subunit activity [89].

Transforming growth factor (TGF)-*β*1 contains similar structure with apoE and lipoproteins containing the apoE3 isoform have higher TGF-*β* levels and bioactivity than those containing apoE4. Association of TGF-*β* with different types of lipoproteins may be beneficial to diseases like atherosclerosis and AD [90].

Intraventricular administration of LPS resulted in higher production of proinflammatory cytokines, along with greater activation of NF*κ*B-regulated genes in the apoE4 than in apoE3 Tg mice [91]. Inflammatory responses of APOE*ε*4 TR mice following intravenous administration of LPS expressing the human APOE*ε*4 allele had higher systemic and brain levels of the proinflammatory cytokines TNF-*α* and IL-6 than their apoE3 counterparts, suggesting an isoform-specific effect of the immunomodulatory properties of apoE [28].

COG1410, an apoE-mimetic peptide, was also associated with a reduction in TNF-*α*, IL-1*β*, IL-6, and IL-12 levels in both apoE3 TR and apoE4 TR mice assessed at 24 hours in the caecal ligation and puncture model of peritonitis [92].

Peripheral blood mononuclear cells (PBMCs) from AD patients with APOE*ε*4 allele produced higher spontaneous and phorbol-12-myristate-13-acetate-(PMA-) induced levels of IL-1*β* after controlling for confounders. APOE4*ε* carriers showed higher concentrations of IL-1*β* than their counterparts without APOE*ε*4 after PMA-stimulation [93]. The APOE*ε*4 allele was associated with lower serum levels of IL-10 in both acute coronary syndrome and chronic stable angina patients [94]. Recombinant apoE suppresses chemokine (C–C motif) ligand (CCL)2 and vascular endothelial growth factor (VEGF) expression in retinal pigment epithelium (RPE) cells in an isoform-dependent manner (apoE4 > apoE3) [95]. The exact molecular mechanisms by which apoE isoforms differentially alter the immune responses have been postulated to influence different signaling pathways. ApoE isoforms might be in part responsible for the differential modulation of NF*κ*B and MAPK pathways [89, 91, 96]. 

A significant interaction between APOE genotype and the response to 17*β*-estradiol was observed for NO and TNF-*α* production from microglia activated by recombinant mouse IFN-*γ* plus either LPS or PIC. The antiinflammatory activity of 17*β*-estradiol is pronouncedly reduced in APOE*ε*4 TR mice compared with APOE*ε*3 TR mice. ApoE genotype may affect the neuroprotective role of 17*β*-estradiol and of hormone replacement therapy on brain function when the APOE*ε*4 gene is expressed [96].

## 6. Effects of Cytokines on the Production of ApoE in Various Tissues

Classical activation of macrophages by proinflammatory cytokines such as IFN-*γ* and TNF-*α* can downregulate apoE production [97–99]. However, antiinflammatory stimuli such as TGF-*β* and estrogen promote apoE synthesis and release [100]. GM-CSF stimulated macrophages had a 3–5-fold reduction in apoE secretion, comparable with that observed for IFN-*γ*, while TNF-*α* and IL-1*β* resulted in a 2-fold decrease. In contrast to the reduction in apoE secretion by these cytokines, TGF-*β* stimulated macrophages secreted 3-fold greater amounts of apoE than controls [100–103].

Treatment of primary rat astrocyte and mixed glial cell cultures with TNF-*α* markedly reduced extracellular apoE protein levels [104]. The effects of apoE in adipose tissue on body lipid homeostasis and atherosclerosis have been widely studied [105]. ApoE is important in modulating triglyceride metabolism in adipocytes. TNF-*α* can suppress the synthesis of apoE in adipocytes [106]. TNF-*α* may activate the binding of NF*κ*B p50 to a binding site at -43 in the apoE promoter. Treatment of adipocytes with TNF-*α* led to increased binding of NF*κ*B p50, decreased binding of p65 and Sp1 to this region of the apoE promoter, and repression of adipocyte apoE gene expression. Reduction of p50 expression using siRNA completely eliminated TNF-*α*-mediated reduction of endogenous apoE gene expression in adipocytes [107].

IFN-*γ* dramatically increased the degradation of intracellular apoE and inhibited the accumulation of apoE in the supernatants of human monocyte-derived macrophages and human acute monocytic leukemia cell line (THP-1) cells via post-translational modification of apoE [97, 108]. IFN-*β*1b has inhibitory effects on the production of apoE mRNA, cellular protein, and secreted protein in blood monocytes [109]. Treatment of primary mixed glial cell cultures with IL-1*β* induced a marked increase of extracellular apoE protein. TGF-*β* can induce the expression of apoE in the THP-1 cell line by activating JNK, MAPK p38 and caseine kinase (CK)2 pathways. Pharmacological inhibitors can prevent this action [110].

## 7. Concluding Remarks

ApoE plays a pivotal role in maintaining the Th1/Th2 balance. Exogenous apoE suppressed the production of proinflammatory cytokines in an isoform-specific manner. Classical activation of macrophages by proinflammatory cytokines can downregulate apoE production and antiinflammatory stimuli can promote apoE synthesis and release, indicating a key role of apoE and cytokines in immunomodulation. ApoE suppressing proinflammatory signalings, and *vice versa*, indicate an intricate apoE-mediated feedback regulation of inflammatory and immune responses.

## Figures and Tables

**Table 1:** The main differences among human apoE isoforms.

Isoform	AA residues		Domain interaction	Binding to LDLR	Lipoprotein-binding preference
	112	158			
ApoE 2	Cysteine	Cysteine	No	Low affinity	HDL
ApoE 3	Cysteine	Arginine	No	High affinity	HDL
ApoE 4	Arginine	Arginine	Yes	High affinity	LDL

AA = amino acid.

LDLR = low density lipoprotein receptor.
